# Fluctuation induced conductivity and pseudogap state studies of Bi_1.6_Pb_0.4_Sr_2_Ca_2_Cu_3_O_10+δ_ superconductor added with ZnO nanoparticles

**DOI:** 10.1038/s41598-021-83218-9

**Published:** 2021-02-22

**Authors:** Ali Aftabi, Morteza Mozaffari

**Affiliations:** 1 Department of Physics, Faculty of Science, University of Kurdistan, 66177-15175 Sanandaj, Iran; 2 Department of Physics, Faculty of Physics, University of Isfahan, 81746-73441 Isfahan, Iran

**Keywords:** Superconducting properties and materials, Superconducting properties and materials, Nanoparticles, Ceramics, Composites

## Abstract

The major limitations of the Bi_1.6_Pb_0.4_Sr_2_Ca_2_Cu_3_O_10+δ_ superconductor are weak flux pinning capability and weak inter-grains coupling that lead to a low critical current density and low critical magnetic field which impedes the suppleness of this material towards practical applications. The addition of nanoscales impurities can create artificial pining centers that may improve flux pinning capability and intergranular coupling. In this work, the influences of ZnO nanoparticles on the superconducting parameters and pseudogap properties of the Bi_1.6_Pb_0.4_Sr_2_Ca_2_Cu_3_O_10+δ_ superconductor are investigated using fluctuation induced conductivity analyses. Results demonstrate that the ZnO nanoparticles addition improves the formation of the Bi_1.6_Pb_0.4_Sr_2_Ca_2_Cu_3_O_10+δ_ phase significantly. Various superconducting parameters include coherence length along c-axis (ξ_c_(0)), penetration depth (λ_pd_(0)), Fermi velocity (v_F_), Fermi energy (E_F_), lower and upper critical magnetic fields (B_c1_(0) and B_c2_(0) respectively) and critical current density (J_c_(0)), are estimated for samples with different amounts of ZnO nanoparticles. It is found that the values of the B_c1_(0), B_c2_(0), and J_c_(0) are improved significantly in the 0.2 wt% ZnO added sample in comparison to the ZnO-free sample. The magnitude and temperature dependence of the pseudogap Δ*(T) is calculated using the local pairs model. The obtained values of T_pair_, the temperature at which local pairs are transformed from strongly coupled bosons into the fluctuating Cooper pairs, increases as the added ZnO nanoparticles concentration enhances up to 0.2 wt%. Also, the estimated values for the superconducting gap at T = 0 K (Δ(0)) are decreased from about 26 meV in ZnO-free sample to about 22 meV in 0.2 wt% ZnO added sample and then increases for higher values of additive.

## Introduction

The superconducting pairing mechanism in high-temperature superconductor (HTS) materials remains rather controversial, more than three decades after their discovery^1,2^. It has been clear that the superconductivity mechanism in HTS materials can be understood by investigating the normal state properties of these materials^3–5^. The short coherence length, high anisotropy, and low density of carriers in the HTS materials lead to a progressive deviation of the resistivity ρ(T) curve from the linear metallic-like behavior in the normal state, at a representative temperature T* > T_c_. This deviation is followed by a considerable rounding that is observed above the transition temperature T_c_^6,7^. This deviation from linear behavior indicates thermal fluctuations of Cooper-pairs. These fluctuations are responsible for the finite probability of the Cooper-pairs formation above T_c_^8^. Thermal fluctuations, in turn, can result in an excess conductivity above T_c_. this is called the fluctuation induced conductivity (FIC)^8–10^. FIC provides an opportunity to investigate the superconducting Cooper-pairs fluctuations behavior in a broad range of temperatures above T_c_. The study of the FIC has attracted significant attention in the research of the HTS materials^10–14^. These interesting studies are keys to providing information about microscopic and superconducting parameters of the HTS materials such as Fermi velocity and Fermi energy of charge carriers, coherence length, cross over temperatures, phase relaxation time (lifetime) of fluctuating pairs, critical magnetic fields, and critical current density^6,8,15^. Moreover, theoretical conceptions of the FIC region or Cooper-pairs generation could be examined^16^.

To explain the fluctuation’s effects several models have been proposed. Aslamazov-Larkin (AL), Lawrence-Doniach (LD), Hikami-Larkin (HL), and Maki-Thompson (MT) models^17–20^ are the most appreciated ones. In the FIC region, the fluctuating pairs in many ways demean the same as conventional superconducting Cooper-pairs without the long-range coherence that is named as “short-range phase correlations” and behave according to the Bardeen–Cooper–Schrieffer (BCS) theory^1,11,21,22^. As the temperature is enhanced, due to the thermal fluctuations, the long-range coherence is missed at T_c_^11,23^. According to the theory of the reduced charge carrier density systems, the in-plane coherence length, ξ_ab_(T), which specifies the size of pairs, decreases with the temperature rising^11,24–26^. The bonding energy of the fluctuating Cooper-pairs is inversely proportional to the coherence length, ε_b_ ~ 1/(ξ_ab_)^2^, therefore it noticeably increases with temperature enhancement. Consequently, fluctuating Cooper-pairs should change their properties^11,21,24,25^. Finally, they are converted into so-called strongly bound bosons which satisfy the Bose–Einstein condensation (BEC) theory. So, as temperature enhances, a BCS-BEC transition in HTS materials is predicted, which has been observed experimentally^27–32^. The strongly bound bosons are very short but extremely stoutly coupled pairs. Consequently, they should be local objects (i.e. not interacting with each other) since the distance between the pairs is extremely greater than the size of the pairs. In addition, thermal fluctuations could not destroy them, and consequently, they can form at much higher temperatures than T_c_^33^.

Besides, the study of the FIC in HTS materials is considered as a sufficiently instructive and effective method to study the pseudogap state properties of these materials^2,11,34–36^. Pseudogap state is a unique phenomenon that occurs only in HTS materials and is observed below a representative temperature T* > T_c_^3,37,38^. In the pseudogap state, the normal and superconducting properties have appeared together^39^. This state is described by the redistribution of Fermi surface states at some characteristic temperature T_c_ < T < T*. At T < T*, due to the reasons which have remained unclear, the density of electronic states at the Fermi surface is reduced. Because of this, it is called the pseudogap state^2,30,33,40^. In the pseudogap state, the density of electronic states does not entirely reach zero while in the superconducting gap the density of the electronic states is zero^2^. This is the main difference between the pseudogap and superconducting states. The realization of the pseudogap state could be helpful to the understanding of the superconducting pairing mechanism in high-temperature superconductors materials^1,2,41–44^. It is essential to search for finding superconductors with higher critical transition temperature^2^. Recently the possible interconnections of the FIC temperature dependence in HTS materials with pseudogap are extensively investigated^2,11,28,36,45,46^. Within the framework of the local pairs model^1,2,21,47^, the magnitude and temperature dependence of pseudogap can be calculated based on the FIC analyses above T_c_^4,5,11,29,36,48,49^.

Among different HTS materials, Bi_1.6_Pb_0.4_Sr_2_Ca_2_Cu_3_O_10+δ_ ((Bi, Pb)-2223) phase, due to the remarkably high T_c_ (~ 110 K), larger values of the critical current density J_c_ and critical magnetic field B_c_, has attracted numerous attention as a promising superconducting material to synthesize different kinds of samples such as wires, thin films, tapes, single crystals, and bulks for large-scale and high-current applications^50–56^. Synthesizing of the (Bi, Pb)-2223 phase is a complex procedure and there is a huge difficulty in the preparation of the single-phase samples since alongside (Bi, Pb)-2223 phase, Bi_1.6_Pb_0.4_Sr_2_CaCu_2_O_8+δ_ ((Bi, Pb)-2212) and Bi_1.6_Pb_0.4_Sr_2_CuO_6+δ_ ((Bi, Pb)-2201) superconducting phases, with a transition temperature of 95 and 20 K respectively, form besides of non-superconducting phases^54,55^. The major limitations of the (Bi, Pb)-2223 phase which impedes the suppleness of this material towards practical applications, are weak flux pinning capability and weak inter-grains coupling^15^. Also, the residual secondary (Bi, Pb)-2212 and (Bi, Pb)-2201 phases which are located at grain boundaries lead to the weak intergranular coupling and preventing the super-current flow^15,55,57^. Under equilibrium conditions, magnetic flux penetrates into the bulk of type-II superconductors above the lower critical field B_c1_ for many high-field materials. At B > B_c1_, this magnetic flux exists in the form of a hexagonal lattice of quantized vortex lines^58^. The vortices strongly interact with each other forming highly correlated configurations such as the vortex lattice^59^. Each vortex is a tube of the radius of the London magnetic penetration depth λ_pd_(T), in which screening currents circulate around a small non-superconducting core of radius ξ(T). The flux produced by screening currents in a vortex equals the flux quantum Φ_0_ = 2.07 × 10^−15^ Wb, so the vortex density n = B/Φ_0_ is proportional to the magnetic induction B. Bulk superconductivity is destroyed when the normal cores overlap at the upper critical field B_c2_ = Φ_0_/2πξ^2^^58^. Besides, a Lorentz force F_L_ = J × B acts on the quantized vortices when a current J is injected into a superconductor. The critical current density J_c_ (T; B) is then defined by the balance of the flux pinning and Lorentz force J_c_(T;B)B = F_p_(T;B), where F_p_ is the volume pinning force produced by pinning defects in the strongly interacting array of flux lines^58,60^. Growth of the structure of the vortex lattice in weakly pinned high-T_c_ superconductors is of paramount importance since it determines superconducting properties that are directly suitable for applications^59^. Inserting the nano-structure phases into the HTS materials is a radical solution to increase flux pinning force^60^. These nano-structure phases can work as prominent flux trapping centers to stop the vortex motion. It has been observed that the addition of suitable amounts of nanostructures to the HTS materials could improve the microstructure, intergranular coupling, flux pining capability, and other superconducting properties of these compounds^6,7,10,61–63^. Since the size of nanoparticles is lower than the penetration depth λ_pd_ and higher than the coherence length ξ, it can lead to a strong interaction between the nanoparticles and flux line network and consequently improved the flux pining capability^64^. There are some reports on the investigation of the effects of different nanoparticles addition on the superconducting properties of the (Bi, Pb)-2223 phase^7,15,16,54,65^.

In the current study, the impacts of ZnO nanoparticles (NPs) on the fluctuation induced conductivity of the (Bi, Pb)-2223 phase have been investigated. The important superconducting parameters were deduced using the FIC analyses. Moreover, the effects of the ZnO NPs on the magnitude and temperature dependence of the pseudogap in (Bi, Pb)-2223 phase were studied.

## Methods

To investigate the effects of ZnO NPs on the microscopic and superconducting parameters of the (Bi, Pb)-2223 phase, a series of composite samples of Bi_1.6_Pb_0.4_Sr_2_Ca_2_Cu_3_O_10+δ_ /(ZnO)x with 0.0 ≤ x ≤ 1 wt% (x = 0.0, 0.1, 0.2, 0.3, 0.5 and 1 wt%) were prepared by conventional solid-state reaction method. Appropriate stoichiometric quantities of high-purity Bi_2_O_3_, Pb_3_O_4_, SrCO_3_, CaCO_3_, and CuO powders (all of the analytical grades with minimum purities of 99.9%) were used as raw materials. As a first step, the raw materials were mixed and grounded in an agate mortar for about 1.5 h to ensure homogeneity. The mixed materials were calcined at 820 °C for 24 h in the air followed by furnace cooling to room temperature. Then calcinated materials reground into a fine powder. The calcining and grinding procedures were repeated three times. In the next step, different amounts of ZnO NPs (0.0 to 1 wt%) with a mean crystallite size of 15 ± 2 nm were added to the calcined powder and ground in agate vials for 2 h using a planetary ball mill (FRITCH P7). The mixed powder samples were pressed into blocks (15 mm × 4 mm and about 2 mm in height), under the pressure of 7 tons/cm^2^. In the final step, the composite samples were sintered at 810 °C in the air for 120 h.

The phase identification of the prepared samples was carried out by powder X-ray diffraction using a Bruker D8 Advance X-ray diffractometer, with Cu Kα radiation (λ = 1.506 Å) in the diffraction angle range of 3° ≤ 2θ ≤ 80°. The concentrations of the (Bi, Pb)-2223 and (Bi, Pb)-2212 phases formation were estimated from the X-ray diffraction (XRD) peaks intensities. The microstructure and grain morphology of the various composites have been recognized by a scanning electron microscope (SEM) (Philips, XL30 model).

To investigate the superconducting properties, fluctuation induced conductivity, and pseudogap properties, the temperature dependence of the resistivity ρ(T) was measured by the conventional four-point-probe technique. For fluctuation induced conductivity analysis, Aslamazov-Larkin and Lawrence-Doniach models were employed to estimate the fluctuation dimensionality, zero coherence length along c-axis (ξ_c_ (0)), penetration depth (λ_pd_(0)) Fermi velocity (v_F_), Fermi energy (E_F_), lower and upper critical magnetic fields (B_c1_(0) and B_c2_(0)), and critical current density J_c_(0). To measure critical current density, the V-I curves of the different samples have been recorded at 77 K by the four-point-probe technique. Also, to calculate the magnitude and temperature dependence of the pseudogap, the local pairs model has been used.

## Results and discussion

### XRD analysis and phase formation investigation

**Figure 1:**
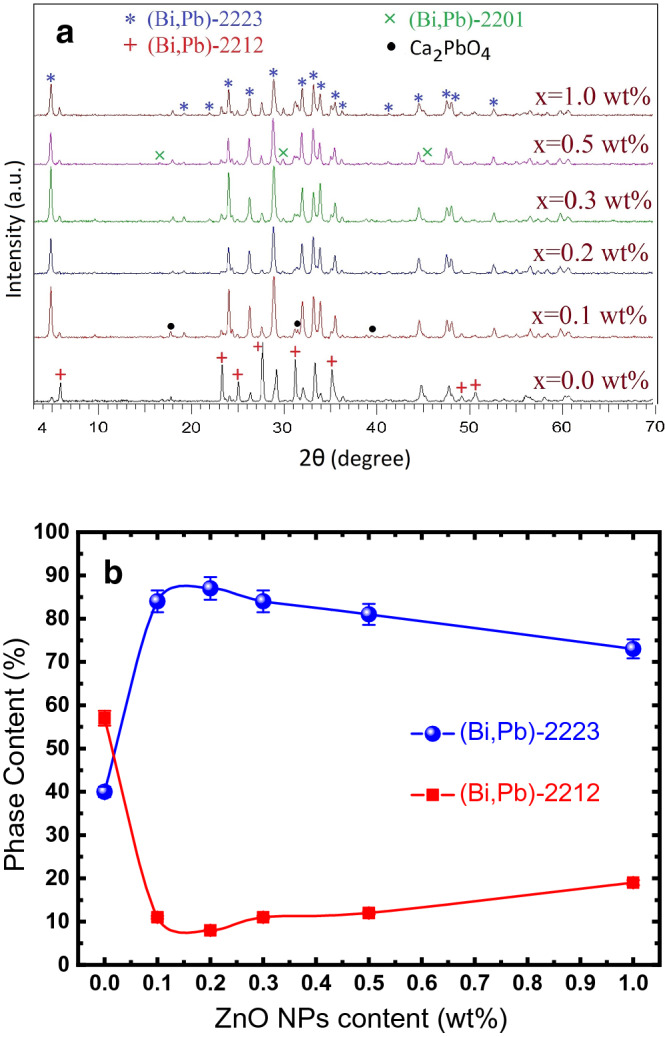
(**a**) XRD patterns of the sintered (Bi,Pb)-2223/(ZnO NPs)x superconducting composites with x = 0.0, 0.1, 0.2, 0.3, 0.5 and 1.0 wt%. (**b**) Variations of the (Bi, Pb)-2223 and (Bi, Pb)-2212 phases volume fractions as a function of the ZnO NPs content for sintered composites. The solid lines are to guide the eye.

XRD patterns of the (Bi, Pb)-2223/(ZnO NPs)x (x = 0.0, 0.1, 0.2, 0.3, 0.5 and 1 wt%) composites are displayed in Fig. 1a. The pattern of the ZnO-free sample (x = 0.0 wt%) indicates that the major peaks in this sample are related to the (Bi, Pb)-2212 and (Bi, Pb)-2223 phases and some weak peaks are observed which belong to Ca_2_PbO_4_ and (Bi, Pb)-2201 as minor phases. It shows that (Bi, Pb)-2212 is the dominant phase in this sample. As can be observed, the (Bi, Pb)-2223 peaks’ (2θ = 5°, 24°, 26.5°, 29°, 32°, and 34°) intensity for the ZnO-added samples enhanced significantly and (Bi, Pb)-2212 peaks’ (2θ = 6°, 23.5°, 25, 27.5°, 31.5°, 35.5°, and 50.5°) intensity dropped in comparison with the ZnO-free sample. The samples with ZnO additive are composed of (Bi, Pb)-2223 with tetragonal structure as the dominant phase. The relative volume fractions of the (Bi,Pb)-2223 to (Bi,Pb)-2212 phases were estimated from the XRD peaks’ intensities as described in Supplementary Note 1. The calculated relative volume fractions of (Bi, Pb)-2223 and (Bi, Pb)-2212 phases versus added ZnO NPs concentrations are demonstrated in Fig. 1b. As seen, by increasing the ZnO concentration, the (Bi, Pb)-2223 phase volume fraction increases from ~ 38 wt% in the ZnO-free sample to ~ 87 wt% in the sample with x = 0.2 wt% and then shows a decreasing behavior for higher values of added ZnO NPs until it reaches to ~ 73 wt% in the sample with x = 1 wt%. On the other hand, the volume fraction of (Bi, Pb)-2212 phase reduces from ~ 58 wt% for the ZnO-free sample to ~ 8 wt% for the sample with x = 0.2 wt% and then enhances for the samples with higher values of ZnO NPs concentrations and reaches to about ~ 19 wt% in the sample with x = 1 wt%. The XRD results indicated that the small amount of the ZnO NPs improves the formation of (Bi, Pb)-2223 phase significantly, which can be attributed to the grain connectivity improvement by ZnO NPs^15^. It has been reported that the addition of impurities decreases the partial melting point of the Bi-Sr-Ca-Cu–O system^66^. It is well established that, the optimum sintering temperature is defined just below the partial melting temperature^67^. Consequently, enhancement of (Bi, Pb)-2223 phase formation by adding the ZnO NPs can be attributed to the improvement of the sintering process in added samples. Moreover, as the added ZnO NPs concentration increases, no detectable shifts in (Bi, Pb)-2223 related XRD peaks were observed. This indicates that ZnO NPs are dispersed at the grains’ boundaries and do not change the host crystal structure of the (Bi, Pb)-2223 superconductor phase.

### Morphology and microstructure

**Figure 2:**
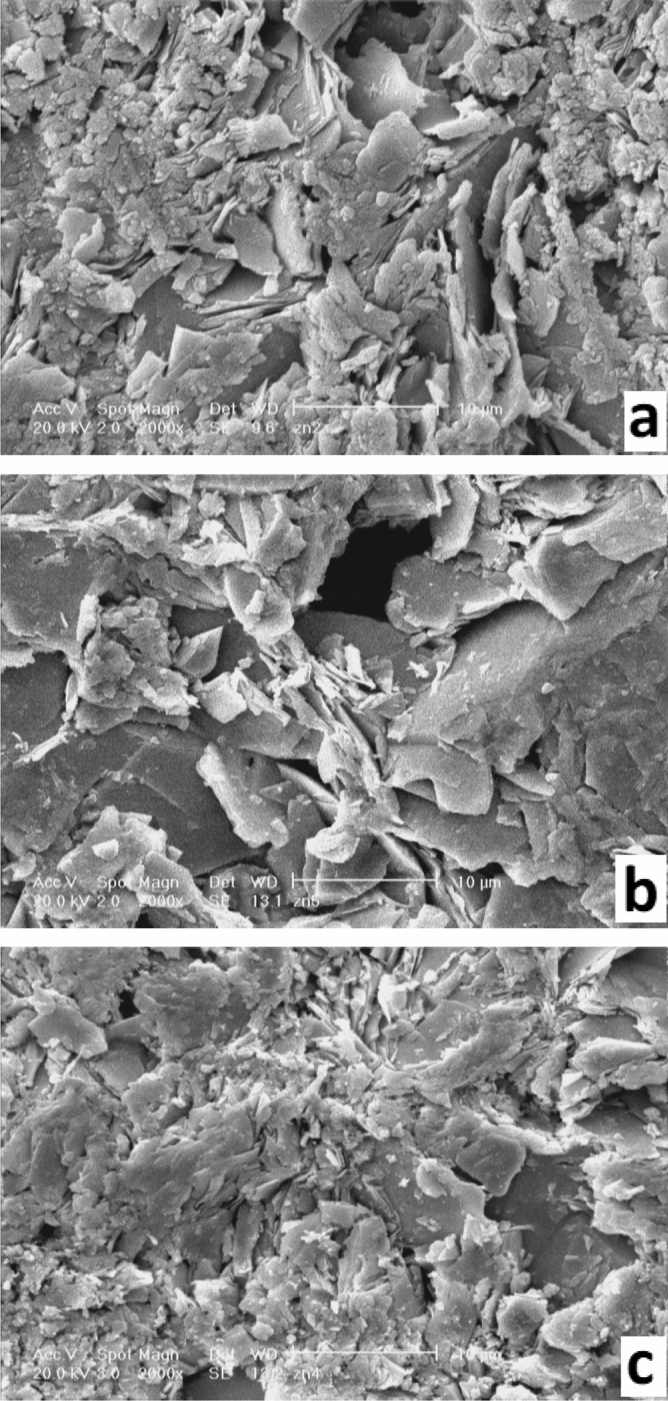
SEM micrographs of the (Bi, Pb)-2223/(ZnO NPs)x composite samples with (**a**) x = 0.0, (**b**) x = 0.2 and (c) x = 1.0 wt%.

The SEM micrographs of the (Bi_,_ Pb)-2223/(ZnO NPs)x composites with x = 0.0, 0.2, and 1 wt%, as typical ones, are represented in Fig. 2a–c respectively. These micrographs show a granular structure that is a typical structure of HTS materials. Also, plate-like grains are observed that are a sign of (Bi, Pb)-2223 phase formation. Compering of the SEM micrographs shows that the concentration and the size of the plate-like grains are enhanced in the samples with ZnO NPs additive compare to the ZnO-free sample. The added samples have coarser grains and lower porosity. These results indicate a better intergranular coupling in the added samples. In addition, the SEM micrographs display that the size and concentration of the plate-like grains are decreased in the sample with x = 1.0 wt% in comparison with the sample with x = 0.2 wt%. These effects are related to the influence of the ZnO NPs on the sintering process. The obtained results from SEM micrographs are in good agreement with the XRD results.

### Electrical resistivity measurements

**Figure 3:**
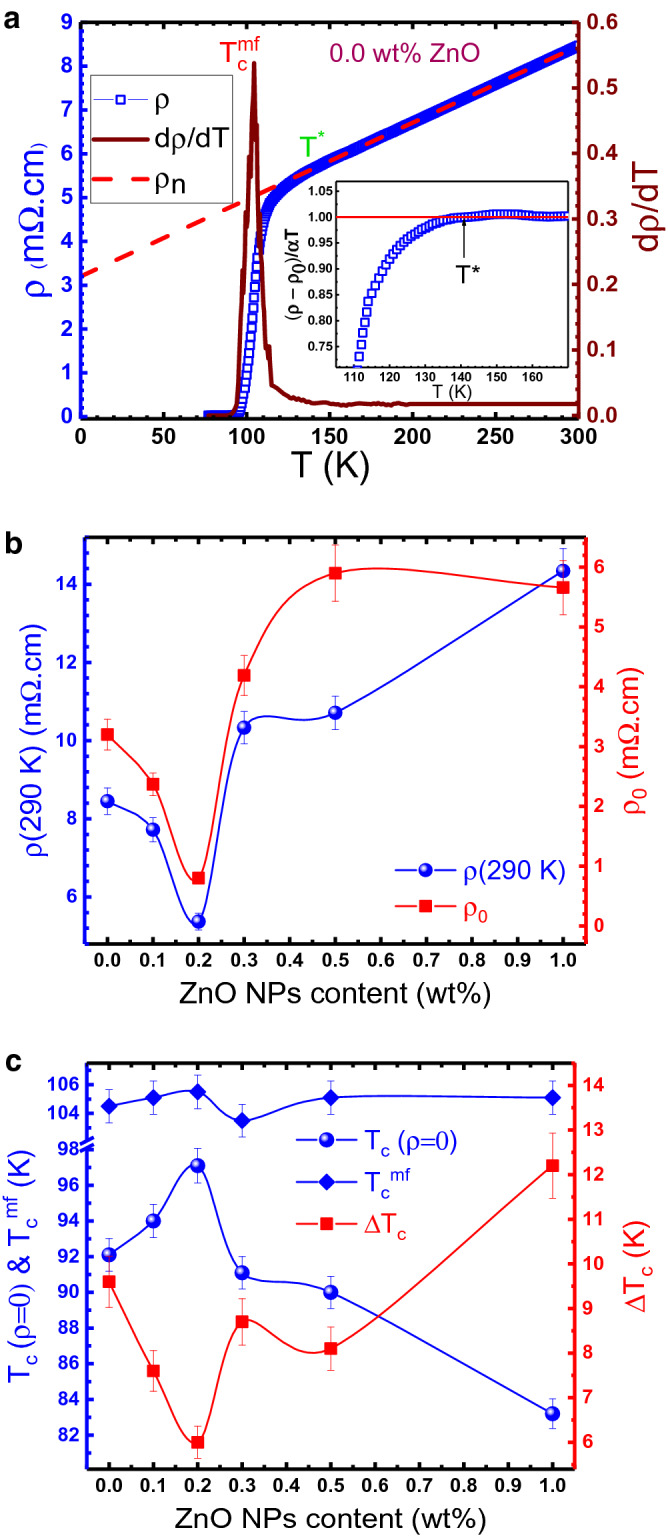
(**a**) Plots of the temperature dependence of electrical resistivity ρ(T), it’s corresponding derivative dρ/dT, and extrapolated normal state resistivity ρ_n_(T) to 0 K, for the sample with x = 0.0 wt%. The inset graph displays a more accurate determination of T* using the criterion (ρ(T)-ρ_0_)/αT = 1. (**b**) Variations of the room temperature resistivity ρ(290 K) and extrapolated zero temperature resistivity ρ_0_ as a function of the ZnO NPs content. (**c**) The mean-field transition temperature T_c_^mf^, zero resistance transition temperature T_c_(ρ = 0), and transition temperature width ΔT_c_ as a function of the ZnO NPs content.

The temperature dependence of resistivity ρ(T), it’s corresponding derivative dρ/dT, and extrapolated normal state resistivity ρ_n_(T), for the ZnO free sample, as a typical one, has been shown in Fig. 3a. The related curves for other samples are represented in Supplementary Fig. S1. As can be seen, for all samples electrical resistivity measurements demonstrate a well-defined metal-like behavior (normal state) followed by a transition to the superconducting state (zero resistance). The normal state resistivity is characterized by the stability of the Fermi surface^2^ and determined from the linear fitting of ρ(T) curve, in the range $2T_{c} \le T \le 300\,{\text{K}}$, according to the Anderson and Zou relationship^68^:

1 $$\uprho _{\text{n}} \left( {\text{T}} \right) =\uprho _{0} + \upalpha {\text{T,}}$$

where ρ_0_ is the residual resistivity that is specified by extrapolation of the linear fitting to 0 K and α is the temperature coefficient of resistivity. ρ_0_ is temperature independent and indicates the defect density and homogeneities of the samples while α is a temperature-dependent intrinsic parameter. For temperatures above T*, resistivity decreases linearly with temperature by a gradient α = dρ/dT. Below T* progressive deviance of the resistivity curve from the linear behavior is observed that followed by a notable rounding that displays the appearance of fluctuation induced conductivity. Some electron pairs start to appear as the temperature is decreased below T*. These electron pairs lose the long-range coherence needed for superconductivity. By further temperature decreasing, the number of formed electron pairs improves until achieved the mean-field critical temperature ${\text{T}}_{\text{c}}^{\text{mf}}$, where all conducting electrons are paired and act in correlation^7^. As shown in Fig. 3a, the ${\text{T}}_{\text{c}}^{\text{mf}}$ can be determined using the derivative of the resistivity curve dρ/dT. It is the temperature corresponding to the maximum in the plot of dρ/dT versus temperature. Also, the transition temperature width (ΔT_c_) was calculated by measuring the full width at half the maximum of the dρ/dT curve. To more accurate determination of T*, the criterion $\left( {{\uprho }_{\text{n}} \left( {\text{T}} \right) - {\uprho }_{0} } \right)/{\alpha T} = 1$ was used^36^. The plot of $\left( {{\uprho }_{\text{n}} \left( {\text{T}} \right) - {\uprho }_{0} } \right)/{\alpha T}$ against T, is shown in the inset of Fig. 3a. T* is defined as a temperature which the $\left( {{\uprho }_{\text{n}} \left( {\text{T}} \right) - {\uprho }_{0} } \right)/{\alpha T}$ deviates from 1.

The different parameters include ρ(290 K) (room temperature resistivity), ρ_0_, T_c_(ρ = 0) (zero-resistivity critical temperature), ΔT_c_, ${\text{T}}_{\text{c}}^{\text{mf}}$ , α and T*, were calculated from ρ(T) curves for various composites. Figure 3b shows the variation of ρ(290 K) and ρ_0_ versus ZnO NPs content. As observed, both ρ(290 K) and $\rho_{0}$ decrease with the increasing of the ZnO NPs concentration up to 0.2 wt%. The values of the ρ(290 K) and ρ_0_ in the sample with 0.2 wt% ZnO are reached close to 0.63 times and 0.25 times of the ZnO-free sample values, respectively. The obtained values of ${\text{T}}_{\text{c}}^{\text{mf}}$, T_c_(ρ = 0) and ΔT_c_ for different samples are illustrated in Fig. 3c. As can be seen, ${\text{T}}_{\text{c}}^{\text{mf}}$ is almost constant (about 105 K) for different x values. Moreover, T_c_(ρ = 0) increases from 92 K for the ZnO-free sample to 97 K in the 0.2 wt% added sample and then decreases for samples with higher ZnO NPs concentrations and reaches to 83 K in the sample with x = 1.0 wt%. Enhancement of the T_c_(ρ = 0) is attributed to the improvement of the inter-grain coupling by adding ZnO NPs. Figure 3c also shows that transition temperature width (ΔT_c_) is decreased from about 9.5 K in the ZnO-free sample to about 5.9 K in the sample with x = 0.2 wt% and then increased for higher values of the ZnO NPs concentrations. The reduction of the ΔT_c_ is related to the improvement of the homogeneity by adding ZnO NPs up to 0.2 wt%. The α and T* values for different samples are recorded in Table 1. As it can be seen, there is not a regular trend for α and T* values with increasing the ZnO NPs concentration. The results show that the maximum T* and α values are related to the x = 0.3 and 1.0 wt% respectively.

**Table 1:** Conductivity exponents, crossover `oscopic parameters estimated from electrical resistivity measurements and fluctuation induced conductivity analyses.

x (wt%)	α (μΩ.cm/K)	T* (K)	λ_CR_	λ_3D_	λ_2D_	λ_SW_	T_G_ (K)	T _3D-2D_ (K)	T_2D-SW_ (K)	ΔT_3D_ (K)	ΔT_2D_ (K)	N_G_ × 10^−2^	κ
0.0	17.5	143.0	0.33	0.64	1.42	3.02	105.9	108.1	120.4	2.2	12.3	1.40	72.10
0.1	17.9	156.1	0.32	0.67	1.21	2.91	106.4	107.9	126.4	1.5	18.5	1.29	75.11
0.2	15.3	147.0	–	0.62	1.33	2.93	106.1	107.8	126.9	1.7	19.1	0.57	64.27
0.3	20.5	175.1	0.25	0.62	1.35	3.21	105.4	106.1	139.6	0.7	33.5	1.87	84.55
0.5	16.1	159.0	0.27	0.60	1.03	2.87	106.5	108.0	126.3	1.5	18.3	1.33	74.98
1.0	29.0	161.0	0.28	0.64	1.38	2.95	106.8	109.5	138.0	2.7	28.5	1.66	71.59

These results indicate that by adding a small amount of ZnO NPs induced rectification of the inter-grain nature by reducing the (Bi, Pb)-2212 phases. The (Bi, Pb)-2212 phase on the grain boundaries acts as a weak links consequently reduces the intergranular coupling. Thus, decreasing the (Bi, Pb)-2212 phase and increasing the (Bi, Pb)-2223 phase improve the transport properties of the samples with x = 0.1 and 0.2 wt% ZnO NPs additive. Therefore, the decreases in the ρ(290 K) and ρ_0_ and increases in the T_c_(ρ = 0) are related to the improvement of the intergranular coupling. At higher additive concentrations, in addition to enhancement of the (Bi, Pb)-2212 phase, the ZnO NPs are believed to sit at grain boundaries and create weak links between superconducting grains, resulting in enhancement of the ρ_0_ and ρ(290 K) and reduction of the T_c_(ρ = 0).

### Fluctuation-induced conductivity

The excess conductivity (Fluctuation induced conductivity) Δσ is defined as a difference between measured conductivity σ(T) and the normal state conductivity σ_n_(T) extrapolated to the low T region^69^. It can be calculated as:

2 $$\Delta {\upsigma } = {\upsigma }\left( {\text{T}} \right) - {\upsigma }_{\text{n}} \left( {\text{T}} \right) = \frac{1}{{\uprho }\left( {\text{T}} \right)} - \frac{1}{{\uprho }_{\text{n}} \left( {\text{T}} \right)},$$

where ρ(T) is the measured resistivity and ρ_n_(T) is the normal state resistivity extrapolated to the low T region. The Aslamazov-Larkin (AL) model^17^ and Lawrence-Doniach (LD) model^18^ were used for fluctuation induced conductivity analyses. According to the AL model^17^, the fluctuation induced conductivity region consists of three different regimes include critical, mean-field, and short-wave fluctuations. That the mean-field regime comprises of three different parts indicating three-dimensional (3D), two-dimensional (2D), and one-dimensional (1D) fluctuations regimes. According to this model, the excess conductivity is written as:

3 $$\Delta\upsigma = {\text{C}}\upvarepsilon ^{ -\uplambda } ,$$

where ${\upvarepsilon } = \frac{{\text{T}} - {\text{T}}_{\text{c}}^{\text{mf}} }{{\text{T}}_{\text{c}}^{\text{mf}} }$ is a reduced temperature, and λ is a critical exponent related to the conduction dimensionality which has values of 0.3, 0.5, 1, 1.5, and 3 respectively for critical (CR), three-dimensional (3D), two-dimensional (2D), one-dimensional (1D), and short-wave (SW) fluctuations regimes. C is the temperature-independent fluctuation amplitude which is given in 1D, 2D, and 3D fluctuations by the following equations:

4 $${\text{C}} = \left\{ {\begin{array}{*{20}l} {\frac{{\text{e}}^{2} }{32\hbar {\upxi }_{\text{c}} \left( 0 \right)}} \hfill & {for\,3D\,fluctuation} \hfill \\ {\frac{{\text{e}}^{2} }{16\hbar {\text{d}}}} \hfill & {for\,2D\,fluctuation} \hfill \\ {\frac{{{\text{e}}^{2} {\upxi }_{\text{c}} \left( 0 \right)}}{{32\hbar {\text{s}}}}} \hfill & {for\,3D\,fluctuation} \hfill \\ \end{array} } \right.,$$

where e is the charge of the electron, ℏ is the reduced Planck’s constant, ξ_c_(0) stands for zero-temperature coherence length along the c-axis, d displays the effective layer thickness of the 2D system and *s* presents the cross-sectional area of the 1D system.

The AL theory was modified by Lawrence and Doniach (LD) for polycrystalline and layer superconductors^18^. In cuprate superconductors, superconductivity takes place principally in 2D CuO_2_ planes which are coupled by Josephson tunneling. In the LD model excess conductivity is expressed as:

5 $$\Delta\upsigma = \frac{{\text{e}}^{2} }{16{\hbar }{\text{d}}} \left( {1 + {\text{J}}\upvarepsilon ^{ - 1} } \right)^{ - \frac{1}{2}}\upvarepsilon ^{ - 1} ,$$

where ${\text{J}} = \left( {2{\upxi }_{\text{c}} \left( 0 \right)/{\text{d}}} \right)^{2}$ represents inter-layer coupling strength. For the strong coupling (J >  > 1) the above equation reduces to the 3D fluctuations condition in Eq. (5) (λ = 0.5) and for the weak coupling (J <  < 1) we get the 2D fluctuation condition in Eq. (5) (λ = 1). The following expression results for cross-over temperature between 3 and 2D fluctuation regimes

6 $${\text{T}}_{3{\text{D}} - 2{\text{D}}} = {\text{T}}_{\text{c}}^{\text{mf}} \left[ {1 + \left( {2{\upxi }_{\text{c}} \left( 0 \right)/{\text{d}}} \right)^{2} } \right].$$

The excess conductivity Δσ(T) was calculated according to Eq. (2). The ln-ln plots of the Δσ versus the reduced temperature ε for different samples are presented in Supplementary Fig. S2. Plots display the existence of three regions include critical, mean-field, and short-wave fluctuation regions. The mean-field region of each sample consists of two distinct linear parts comprising 3D and 2D fluctuations regimes. The various regions of the curves were linearly fitted and the values of the conductivity exponent λ were obtained from the slopes. The different regions are separated from each other by crossover temperatures. The obtained values of the conductivity exponent λ and crossover temperatures for various samples are demonstrated in Table 1.

The short-wave region is the first sector which lies at temperatures much higher than the ${\text{T}}_{\text{c}}^{\text{mf}}$. In this region, the fluctuation induced conductivity reduces sharply with λ_SW_ ≈ 3 (Table 1). Also, the characteristic wavelength of the order parameter becomes comparable to the coherence length order^7^. These behaviors are related to the variation in the density of carriers or the band structures in the Fermi surface, where both of them have a major influence on the change in the order parameter^70,71^. By decreasing the temperature, a transition from the short-wave region to the 2D fluctuation region occurs at temperature T_2D-SW_. As shown in Table 1, the 2D conductivity exponent λ_2D_ values vary between 1.03 and 1.42. In this region, the charge carriers move along the CuO_2_ planes and conductivity mainly occurs from charge carriers limited in the CuO_2_ layers^52^. By further temperature reduction close to ${\text{T}}_{\text{c}}^{\text{mf}}$, the conductivity exponent decreases, and the 3D fluctuation region starts at T_3D-2D_. For different samples, the values of the λ_3D_ changes between 0.60 and 0.64 (Table 1). In this region, the charge carriers cross the barrier layers to reach the conducting CuO_2_ layers. They move between the planes and more influenced by thermal fluctuations compared to the 2D region. This implies that the charge carriers tend to move more freely in the whole sample before the Cooper-pairs formation^6^. As shown in Table 1, by increasing the amount of ZnO nanoparticles the width of the 2D fluctuations region ΔT_2D_ increases and reaches to its maximum value in the sample with 0.3 wt% ZnO NPs, whereas the width of the 3D fluctuation region ΔT_3D_ decreased and in the sample with x = 0.3 wt% is minimum.

The final region is the dynamic critical region, where the fluctuations of the order parameter become comparable to the magnitude of the order parameter itself^7^. The Ginzburg–Landau theory breaks down and interaction between Cooper-pairs is assumed^52^. Crossing between the 3D region and the critical region occurs at T_G_. The values of λ_CR_ vary between 0.25 and 0.33 (Table 1). These values are in good agreement with the theoretical prediction of the 3D-xy universality class, with dynamics given by the representative E-model^72,73^. Despite of numerous investigations of superconducting fluctuations in the HTS materials, the conclusions about the effects of inhomogeneities which occur at varying length scales, are contradictory. Indeed, some studies have shown that inhomogeneities crucially influence the width of the critical and the mean-field (2D and 3D) regions of superconducting fluctuation^74–77^. It has been observed^74^ that by adding nanoparticles to the (Cu_0.5_Tl_0.5_)Ba_2_Ca_2_Cu_3_O_10-δ_ the critical region is disappeared in some nanoparticle concentrations. Also, it has been reported^77^ that the width of the 3D region was reduced by the addition of nanoparticles in polycrystalline (Bi, Pb)–2223 superconducting matrix, which was explained based on the scattering of mobile carriers across the insulating nanoparticles present at the grain-boundaries.

**Figure 4:**
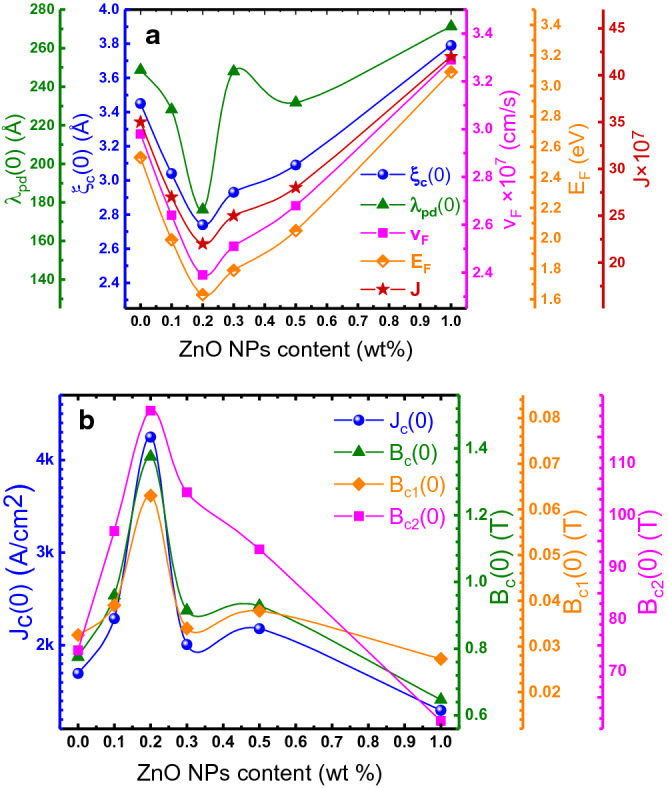
Variations of the estimated values for (**a**) critical current density J_c_(0), thermodynamic magnetic field B_c_(0), lower and upper critical magnetic fields B_c1_(0) and B_c2_(0), (**b**) coherence length along c axis ξ_c_(0), penetration depth λ_pd_(0), Fermi velocity v_F_ and Fermi energy E_F_ of charge carriers and inter-layer coupling strength J, as a function of the ZnO NPs added concentration. The solid lines are to guide the eye.

By the values of the T_3D-2D_ and the LD model (Eq. (6)) the ξ_c_(0) and J were estimated for different samples. Variation of the ξ_c_(0) and J as a function of the ZnO NPs concentration are shown in Fig. 4a. The obtained values for these parameters have a good agreement with reported values for (Bi, Pb)-2223 phase^9,49,52^. The obtained short coherence length (few Å) for prepared samples is generally a trait of the HTS materials and is caused by the presence of the overlapping energy bands^15^. As seen, the ξ_c_(0) and J values decrease as the concentration of ZnO NPs enhances from 0.0 wt% to 0.2 wt%, and then increase for further enhancement of the ZnO NPs concentrations. The minimum value of the ξ_c_(0) for x = 0.2wt% displays the suppression in the density of charge carriers in conducting planes^6,74^.

Moreover, when the ξ_c_(0) value is obtained, the Fermi velocity v_F_ and Fermi energy E_F_ of the charge carriers can be estimated using the following expressions

7 $$ {\text{v}}_{{\text{F}}} = \frac{{5{\pi k}_{{\text{B}}} {\text{T}}_{{\text{c}}} {\upxi }_{{\text{c}}} \left( 0 \right)}}{{2{\text{K}}\hbar }}, $$

8 $${\text{E}}_{\text{F}} = \frac{1}{2}{\text{m}}^{*} {\text{v}}_{\text{F}}^{2} ,$$

where K≈ 0.1242 is a proportionality constant and m* = 10m_0_ is the electron effective mass^14,15^. As displayed in Fig. 4a, it is clear that the Fermi velocity and Fermi energy of the charge carriers are suppressed with increasing in the ZnO NPs concentration from 0.0 to 0.2 wt%. The estimated values of v_F_ are less than that of the free electron (v_F_ = 10^8^ cm/s)^15^. The Fermi velocity depends on the density of carriers in the CuO_2_ planes^78^. As observed, the addition of ZnO NPs from 0 to 0.2wt% reduces the interlayer coupling strength J that suppresses the charge transfer mechanism to the conducting planes, and led to suppressing the density of carriers in the CuO_2_ planes. The obtained Fermi velocity and energy values are comparable with the results obtained by other groups^15,79^.

According to the Ginzburg–Landau (GL) theory^80^, the thermodynamic magnetic critical field B_c_(0) can be calculated form Ginzburg Number (N_G_), which is determined by the equation

9 $$ {\text{N}}_{{\text{G}}} = \left| {\frac{{{\text{T}}_{{\text{G}}} - {\text{T}}_{{\text{c}}}^{{{\text{mf}}}} }}{{{\text{T}}_{{\text{c}}}^{{{\text{mf}}}} }}} \right| = \frac{1}{2}{ }\left[ {\frac{{{\text{k}}_{{\text{B}}} {\text{T}}_{{\text{c}}} }}{{{\text{B}}_{{\text{c}}}^{2} \left( 0 \right){\upgamma }^{2} {\upxi }_{{\text{c}}}^{3} \left( 0 \right)}}} \right]^{2} , $$

where T_G_ represents the crossover temperature from critical to 3D fluctuation regime, k_B_ stands for the Boltzmann’s constant, and γ = ξ_ab_(0)/ξ_c_(0) is the anisotropy parameter with an approximate value around 35 for (Bi, Pb)-2223 system^16^, where ξ_ab_(0) stands for coherence length within the CuO_2_ planes (in the ab plane). The penetration depth λ_pd_(0), lower critical magnetic field B_c1_(0), upper critical magnetic field B_c2_(0), and critical current density J_c_(0) are estimated, after determination of the B_c_(0), using the following GL Eqs. ^6,81^:

10 $${\text{B}}_{\text{c}} = \frac{\Phi _{0} }{2\sqrt 2 \uppi \uplambda _{\text{pd}} \left( 0 \right)\upxi _{\text{ab}} \left( 0 \right)}$$

11 $${\text{B}}_{{\text{c}}1} = \frac{{\text{B}}_{\text{c}} }{{\upkappa }\sqrt 2 }{\text{Ln}}\upkappa$$

12 $${\text{B}}_{{\text{c}}2} = \sqrt 2 {\kappa B}_{\text{c}}$$

13 $${\text{J}}_{\text{c}} = \frac{4{\kappa B}_{{\text{c}}1} }{3\sqrt 3 {\uplambda }_{\text{pd}} \left( 0 \right){\text{Ln}}\upkappa}$$

where ${\Phi }_{0} = {\text{h}}/2{\text{e}}$ is the flux-quantum number and κ = λ_pd_/ξ is the Ginzburg–Landau parameter. As displayed in Fig. 4a, the value of λ_pd_(0) are decreased by the enhancement of ZnO NPs concentration from 0.0 to 0.2 wt% and then increased for higher values of ZnO NPs concentration. The N_G_ parameter are presented in Table 1. The variation of N_G_ with ZnO NPs concentration shows the same trend as λ_pd_(0). The estimated superconducting critical parameters include B_c_(0), B_c1_(0), B_c2_(0), and J_c_(0) are plotted in Fig. 4b. As observed, the critical superconducting parameters B_c_(0), B_c1_(0), B_c2_(0), and J_c_(0) are improved significantly with increasing of the ZnO NPs content to 0.2 wt% and then diminish with further increases in ZnO NPs concentration. Comparing of the obtained critical superconducting parameters for 0.2 wt% ZnO NPs added sample with free-sample shows that, B_c_(0), B_c1_(0), B_c2_(0), and J_c_(0) have been improved by about 78, 100, 58, and 150% respectively. The improvement in these critical parameters is mostly due to the reduction in the magnetic vortices’ motion through improving the flux pining ability inside the composite, revealing the existence of strong pinning sources. Introduced nanoparticles to superconducting matrix plays the role of artificial pinning centers that can improve flux pining capability^6^. The obtained results indicate that the inclusion of ZnO NPs is a promising candidate to reduce the vortices' motion, and improving the critical superconducting parameters in the (Bi, Pb)-2223 phase.

### Critical current density measurements

**Figure 5:**
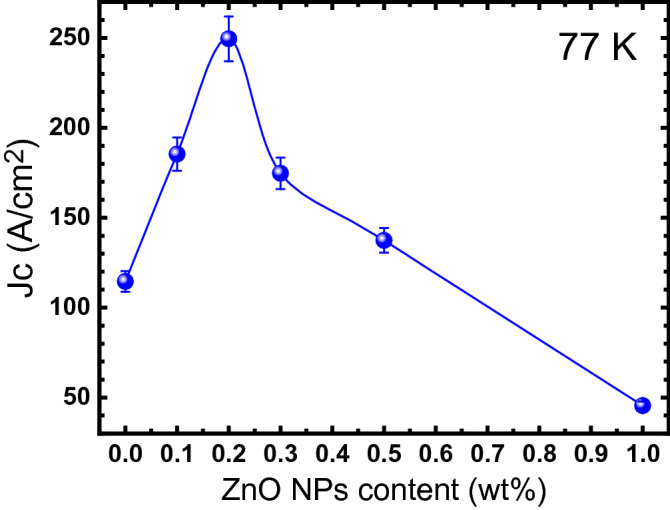
Variations of the measured critical current density, at 77 K , with respect to the ZnO NPs contents.

The measured V-J curves, at 77 K, for different (Bi, Pb)-2223/(ZnO NPs)x composites are demonstrated in Supplementary Fig. S3. Critical current densities of the different composites were determined from the V-J curves, using the criterion of 2 μV/cm. Figure 5 illustrates the measured critical current density as a function of the ZnO NPs added concentration. As observed, it increases from about 114 A/cm^2^ for x = 0.0 wt% to 249 A/cm^2^ for x = 0.2 wt%. For higher values of ZnO NPs, the critical current density decreases and reaches 45 A/cm^2^ for x = 1.0 wt%. As can be seen, the obtained behavior for measured critical current density at 77 K has a good agreement with the estimated one at 0 K, from fluctuation induced conductivity analyses. The improvement of the critical current density by adding the ZnO NPs up to 0.2 wt% is attributed to the improvement of the inter-grain coupling and flux pining capability.

### Pseudogap temperature dependence

As noted above, the pseudogap is a special state of materials that is defined by a reduced electron density of states at the Fermi level. In HTS materials, there is a reduction in the quasi-particle density of states at T < T* (the reasons for this are not entirely discovered), which provides the required conditions for the pseudogap formation^82^. The experimental observation of the pseudogap state in HTS materials has become possible for the first time, primarily due to the development of the angle-resolved photoemission spectroscopy (ARPES) technique^83^. Accordingly, the local pairs model allows us to acquire information about the pseudogap temperature dependence by analyzing the fluctuation induced conductivity^2,11^. In the local pairs model, it is considered that the deviation of the ρ(T) from linearity in the normal state is owing to the opening of the pseudogap at T* > T_c_, leading to the appearance of the excess conductivity, as a consequence of local pairs (strongly coupled bosons) formation^11,36^. These local pairs are subjected to the Bose–Einstein condensate (BEC) theory. When the HTS reaches characteristic temperature T = T*, strongly coupled bosons (SCBs), which do not interact with each other, are starting to form. As the temperature decreases, the concentration of SCBs increases, and some part of them transform into fluctuating Cooper-pairs (FCPs) obey the BCS theory. With more temperature decreasing, the concentration of SCB drops, and the concentration of FCP raises as a result of the transformation of SCB into FCP. At T = T_pair_ all of the SCBs are transferred into the FCPs and in the temperature range, Tc < T ≤ T_pair_ all pairs exist prevailingly in the FCP form^33,48,84,85^.

**Figure 6:**
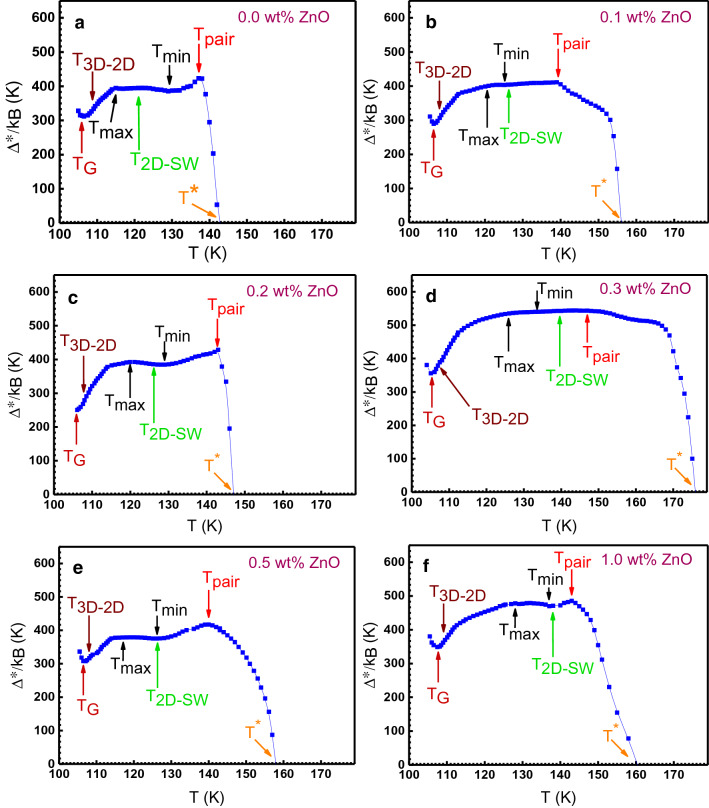
Temperature dependence of the pseudogap Δ*(T) for different (Bi, Pb)-2223/(ZnO NPs)x composites, calculated by local pairs model. The arrows represent characteristic temperatures. The solid lines are to guide the eye.

As noted, the excess conductivity is assumed to appear in the temperature range of T_c_ < T ≤ T* due to the local pairs’ formation and pseudogap opening. This in turn means that the excess conductivity Δσ(T) is a consequence of such processes should enclose information about the magnitude and temperature dependence of the pseudogap^36^. To obtain such information, an equation is required that describes the experimental behaviors of the Δσ(T) over the entire temperature range from T* to T_c_ and would contain the pseudogap parameter explicitly. The local pairs model describes excess conductivity by^11,28,36^

14 $$\Delta\upsigma \left(\upvarepsilon \right) = \frac{{{\text{e}}^{2} {\text{A}}_{4} \left[ {1 - \frac{\text{T}}{{\text{T}}^{*} }} \right]\left[ {\exp \left( { - \frac{{\Delta ^{*} }}{\text{T}}} \right)} \right]}}{{16 \hslash \upxi _{\text{c}} \left( 0 \right)\sqrt {2\upvarepsilon _{\text{co}}^{*} \sinh \left( {\frac{2\upvarepsilon }{\upvarepsilon _{\text{co}}^{*} }} \right)} }},$$

where A_4_ is a numerical coefficient that has the same meaning as the C factor in Eq. (3) and $\varepsilon_{co}^{*}$ is a theoretical parameter that specifies the shape of theoretical curves for T > T_2D-SW_^28,86^. Δ* displays pseudogap and assumed that Δ* = Δ*(T_G_). Besides, (1 − T/T*) determines the number of local pairs formed at T ≤ T* and exp( Δ*/T) determines the number of local pairs destroyed by thermal fluctuations as T approaches T_c_. Solving Eq. (14) for Δ*(T) one can obtain

15 $$\Delta^{*} \,\left( {\text{T}} \right) = {\text{T}}\,{\text{Ln}}\frac{{{\text{e}}^{2} {\text{A}}_{4} \left[ {1 - \frac{\text{T}}{{\text{T}}^{*} }} \right]}}{{\Delta\upsigma \left( {\text{T}} \right) 16 \hslash \upxi _{\text{c}} \left( 0 \right)\sqrt {2\upvarepsilon _{\text{co}}^{*} \sinh \left( {\frac{2\upvarepsilon }{\upvarepsilon _{\text{co}}^{*} }} \right)} }},$$

where Δσ(T) is the experimentally obtained excess conductivity^36^. The mentioned parameters in Eqs. (14) and (15) include ${\upvarepsilon }_{\text{co}}^{*}$, A4, and Δ* are also directly determined from the experiment within the local pairs model as described in Supplementary Note S2. It is assumed that Δ* = Δ*(T_G_) = Δ(0), where Δ(0) is the superconducting gap at T = 0 K^2,87^. The estimated values for prepared samples are recorded in Table 2. The Δ*(T_G_) values for samples are varied between 255 K (22 meV) to 351 K (30 meV). The estimated Δ*(T_G_) values are in good agreement with the reported values of the superconducting gap Δ (0), obtained from the Andreev spectra^49,82,88^. The value of Δ*(T_G_) is dropped from 310 K (26 meV) in the ZnO-free sample to 255 K (22 meV) in the sample with 0.2 wt% additives and then increases to 351 K (30 meV) in the sample with 1.0 wt% ZnO NPs. These behaviors and Δ*(T_G_) values are also in good agreement with experimentally obtained Δ* magnitude at T_G_ (Fig. 6a–f). The magnitude of Δ*(T_G_) was used to determine BCS ratio 2Δ(0)/k_B_T_c_ = 2Δ*(T_G_)/k_B_T_c_ in different samples. As displayed in Table 2, the BCS ratio is about 5.9 in ZnO NPs free sample and drops to 4.8 in the sample with 0.2 wt% ZnO NPs. For higher values of additive, the BCS ratio is increased. The optimal approximation of 2Δ*(0)/k_B_T_c_ for the Bismuth based cuprates is attained at values 5–7^49,89^.

**Table 2:** Parameters of pseudogap analyses using local pairs model.

x (wt%)	α*	$\varepsilon_{co}^{*}$	A_4_	Δ*(T_G_) (K)	$\frac{2\Delta^{*} \left( {T_{G} } \right)}{k_{B} T_{c} }$	T_pair_ (K)	Δ*(T_pair_) (K)
0.0	13.28	0.075	3.90	310	5.9	137	423
0.1	9.92	0.101	4.18	296	5.6	139	411
0.2	9.25	0.108	5.64	255	4.8	143	428
0.3	7.05	0.142	3.65	349	6.7	147	543
0.5	8.72	0.115	1.49	322	6.1	140	417
1.0	6.60	0.151	1.75	351	6.6	143	485

The temperature dependence and magnitude of the pseudogap parameter Δ*(T) have been constructed by Eq. (15) using the obtained values for A_4_, ${\upvarepsilon }_{\text{co}}^{*}$, ξ_c_(0), and T* parameters. The Δ*(T) curves for different samples are demonstrated in Fig. 6a–f. All the curves show the shape typical for HTS materials. As can be seen, by temperature decreasing the pseudogap value first increases and then reduces after passing through a maximum at T = T_pair_. This reduction is due to the variation of the SCBs into the FCPs. With the further temperature decreasing, Δ*(T) usually shows a minimum at T_min_ ~ T_2D-SW_, and then it increases slightly and reaches a maximum at T_max_ ~ T_3D-2D_ followed by a minimum always at T_G_. Below T_G_, there is an abrupt jump in Δ*(T) at T → T_c_. The obtained values of T_pair_ and Δ*(T_pair_) for different samples are shown in Table 2. As seen, T_pair_ is increased from 137 to 147 K by increasing the ZnO NPs concentration from 0.0 to 0.3 wt% and then is decreased for the samples contain more ZnO NPs. Also, the maximum value of the pseudogap Δ*(T_pair_) is varied between 411 K (35 meV) for the sample contains 0.1 wt% ZnO NPs to 543 K (46 meV) for the sample with 0.3 wt% additives. These obtained values have a good agreement with reported values for Bi-(2223) phase^49,82^.

These results show that fluctuation induced conductivity analysis is a sufficiently instructive and effective method to study the pseudogap state properties of HTS materials. Different mechanisms can affect the superconducting properties of the (Bi, Pb)-2223 phase by the addition of ZnO NPs. These mechanisms include intergranular coupling, flux pining capability, and intragranular properties. As observed the addition of ZnO nanoparticles up to 0.2 wt% improves the intergranular coupling by decreasing the undesirable (Bi, Pb)-2212 phase. Besides, the added ZnO nanoparticles can play the role of artificial pinning centers and led to the enhancement in the flux pining capability. Therefore, the improvement in critical current density and critical magnetic fields is attributed to the improvement of the flux pinning capability and inter-grain coupling. On the other hand, as observed, the macroscopic superconducting parameters of the samples (ξ_c_(0), E_F_, v_F_, λ_pd_, and J) were changed by the addition of the different ZnO NPs concentration. It shows that the added ZnO NPs affected the intragranular properties of the samples. Accordingly, the variation of the pseudogap properties can be related to the competition between intergranular coupling, intragranular properties, and flux pining capability.

## Conclusion

The impacts of the ZnO nanoparticles addition on the microscopic, superconducting, and pseudogap properties of the Bi_1.6_Pb_0.4_Sr_2_Ca_2_Cu_3_O_10+δ_ superconductor are investigated using fluctuation induced conductivity analyses. The XRD results show that the inclusion of small amounts of the ZnO NPs improves the (Bi, Pb)-2223 phase formation significantly. The volume fraction of (Bi, Pb)-2223 phase increases from ~ 38 wt% in the pure sample to ~ 87 wt% in the sample with 0.2 wt% additive. As the ZnO NPs concentrations increases, no detectable shifts on the XRD peaks of the (Bi, Pb)-2223 phase are observed that indicates the ZnO NPs dispersed at the grain boundaries by filling the spaces between (Bi, Pb)-2223 grains and then does not change the crystal structure. The SEM micrographs also displayed enhancement of plate-like grains which is a sign of the (Bi, Pb)-2223 phase formation and detects the improvement of the intergranular coupling in ZnO-added samples. Electrical resistivity measurements confirm the improvement of the transport properties and reduction in the room temperature and residual resistivity by increasing the ZnO NPs concentrations up to 0.2 wt%. Also, the zero-resistance critical temperature T_c_(ρ = 0) is enhanced by introducing ZnO NPs and reached its maximum values for the sample with 0.2 wt% ZnO NPs.

The fluctuation induced conductivity analyses were carried out using the Aslamazov-Larkin and Lawrence-Doniach models. The analyses on the prepared composites indicate the existence of four distinct fluctuation regimes include critical, 3D, 2D, and short-wave fluctuations. The dimensionality of the fluctuation regions and the microscopic parameters such as coherence length along the c-axis ξ_c_(0), inter-layer coupling strength J, Ginsberg number N_G_, penetration depth λ_pd_(0), Fermi velocity v_F_, and Fermi energy E_F_ of the charge carriers were estimated. The results show that the width of the 3D fluctuations region is suppressed while the width of the 2D fluctuations region is enhanced by increasing the ZnO NPs concentration up to 0.3 wt%. It is observed that as the added ZnO NPs concentration increases up to 0.2 wt%, the obtained values for ξ_c_(0), J, N_G_, λ_pd_(0), v_F_ and E_F_ decreases and reaches their minimum values and for higher amounts of ZnO NPs they grow up. The superconducting critical parameters include thermodynamic magnetic field B_c_(0), lower and upper critical magnetic fields (B_c1_(0), and B_c2_(0)), and critical current density J_c_(0) were calculated using the Ginzburg–Landau theory. The results demonstrate a significant improvement in these important superconducting parameters. The values of B_c_(0), B_c__1_(0), B_c__2_(0) and J_c_(0) increase about 78, 100, 58, and 150% respectively in the sample with 0.2 wt% additive in comparison with the ZnO NPs free sample. The improvement in these critical parameters is ascribed to the reduction of magnetic vortices motion and improvement of intergranular coupling by the appropriate small content of ZnO NPs inclusion. Since the addition of ZnO NPs develops artificial pinning centers inside the (Bi, Pb)-2223 matrices, hence the magnetic vortices motion is reduced in samples with a small content of ZnO NPs.

Finally, the magnitude and temperature dependence of the pseudogap Δ*(T) was calculated using the local pairs model. The obtained Δ*(T) curves show the shape characteristic for high-temperature superconductors with a maximum at T_pair_ (the temperature at which local pairs are transformed from strongly coupled bosons (SCBs) into the fluctuating Cooper pairs (FCPs)) and a minimum at T_G_ (crossover temperature between critical and 3D fluctuation regions). The results indicate that the value of T_pair_ increases from about 137 K for the ZnO NPs free sample to about 147 K in the 0.3 wt% ZnO NPs added sample. The value of Δ*(T_G_) is dropped from 26 to 22 meV as the added ZnO NPs concentration increases from 0.0 to 0.2 wt% and then increases for higher values of additive and reaches to 30 meV in the sample contains 1.0 wt% ZnO NPs. The Δ*(T_G_) is equal to the superconducting gap at T = 0 K, Δ(0). The obtained values for Δ*(T_G_) was used to determine the BCS ratio of 2Δ(0)/k_B_T_c_ in different samples. The BCS ratio is decreased from 5.9 in the ZnO NPs free sample to 4.8 in the sample with 0.2 wt% ZnO NPs and then enhanced by more increase in the additive concentration.

In conclusion, It is found that the addition of 0.2 wt% ZnO NPs into the (Bi, Pb)-2223 superconducting matrix improves the (Bi, Pb)-2223 phase formation, inter-grain coupling, and flux pinning capability which leads to a significant enhancement in the critical current density and critical magnetic fields.

## Supplementary Information

Supplementary Information.
