# Supplementary material for: Fluctuation induced conductivity and pseudogap state studies of Bi1.6Pb0.4Sr2Ca2Cu3O10+δ superconductor added with ZnO nanoparticles
Source: Sci Rep. 2021 Feb 22;11:4341. doi: 10.1038/s41598-021-83218-9 (PMC7900248; doi:10.1038/s41598-021-83218-9)
Supplement: Supplementary file 1 — Supplementary Information. [file 41598_2021_83218_MOESM1_ESM.pdf]

Fluctuation Induced Conductivity and Pseudogap State Studies of $Bi_{1.6}Pb_{0.4}Sr_2Ca_2Cu_3O_{10+\delta}$ Superconductor Added with ZnO Nanoparticles

Ali Aftabi[1,*], Morteza Mozaffari[2]

[1]Department of Physics, Faculty of Science, University of Kurdistan, Sanandaj, 66177-15175, Iran

[2]Department of Physics, Faculty of Science, University of Isfahan, Isfahan, 81746-73441, Iran

*Corresponding author: a.aftabi@uok.ac.ir & a.aftabi62@gmail.com

Supplementary Note 1:

The relative volume fractions of the (Bi,Pb)-2223 and (Bi,Pb)-2212 phases were estimated from the XRD peak intensities according to the following equations:

$$\left((\mathrm{Bi,Pb})\ 2223\right)\% = \frac{\sum \mathrm{I}((\mathrm{Bi,Pb})\ 2223)}{\sum \mathrm{I}((\mathrm{Bi,Pb})\ 2223)+\sum \mathrm{I}\left((\mathrm{Bi,Pb})\ 2212\right)+\sum \mathrm{I}(\mathrm{other})} \times 100\ , \quad (S1)$$

And

$$\left((\mathrm{Bi,Pb})\ 2212\right)\% = \frac{\sum \mathrm{I}((\mathrm{Bi,Pb})\ 2212)}{\sum \mathrm{I}((\mathrm{Bi,Pb})\ 2223)+\sum \mathrm{I}\left((\mathrm{Bi,Pb})\ 2212\right)+\sum \mathrm{I}(\mathrm{other})} \times 100\ , \quad (S2)$$

where I((Bi,Pb)-2223), I((Bi,Pb)-2212) and, I(other) are intensities of all distinguishable XRD peaks for the corresponding phases respectively.

Supplementary Note 2:

One of the main intrinsic properties of most HTS materials is that in a region at $T > T_{2D\text{-}SW}$ or accordingly $\varepsilon > \varepsilon_{2D\text{-}SW}$ ($Ln(\varepsilon) > Ln(\varepsilon_{2D\text{-}SW})$) the inverse of the excess conductivity is exponentially dependent on ε ($\Delta\sigma^{-1} \sim \exp(\varepsilon)$) [32,33,103]. Consequently, in this region $\mathrm{Ln}(\Delta\sigma^{-1})$ is a linear function of ε with the slope α*. The ε^*_{co} parameter is defined as $\varepsilon^*_{co} = \frac{1}{\alpha^*}$ [103]. To obtain the α* values, $\mathrm{Ln}(\Delta\sigma^{-1})$ were plotted as a function of Ln(ε) for different samples, as shown inset to Fig S4. The slope α* of the linear region above $T_{2D\text{-}SW}$ were estimated by the linear fitting.

To determine A_4, it is necessary to calculate the Ln(Δσ) as a function of Ln(ε) by Eq. (14) in the entire temperature interval $T_c<T<T^*$ and fit it to the experimentally obtained curves in the range of 3D fluctuations region. However, Δ* remains undetermined and it is necessary to finding coefficient A_4. It is assumed that $\Delta^* = \Delta^*(T_G) = \Delta(0)$, where Δ(0) is the superconducting gap at T=0 [2,104]. To estimate $\Delta^*(T_G)$, the experimental values of Ln Δσ must be plotted as a function of 1/T and fitted with Eq. (14). The choice of such coordinates is due to the strong sensitivity of the LnΔσ(1/T) shape to the Δ* value in Eq. (14), which makes it possible to estimate this parameter with high accuracy [59]. To determine the A_4 and $\Delta^*(T_G)$ parameters for various prepared samples, the experimentally obtained Ln(Δσ) versus Ln(ε) and Ln(Δσ) versus 1/T curves were fitted by Eq. (4), using calculated values for T*, $\xi_c(0)$ and ε^*_{co} in previous steps. The related curves for different samples are demonstrated in Figs. S4 and S5.

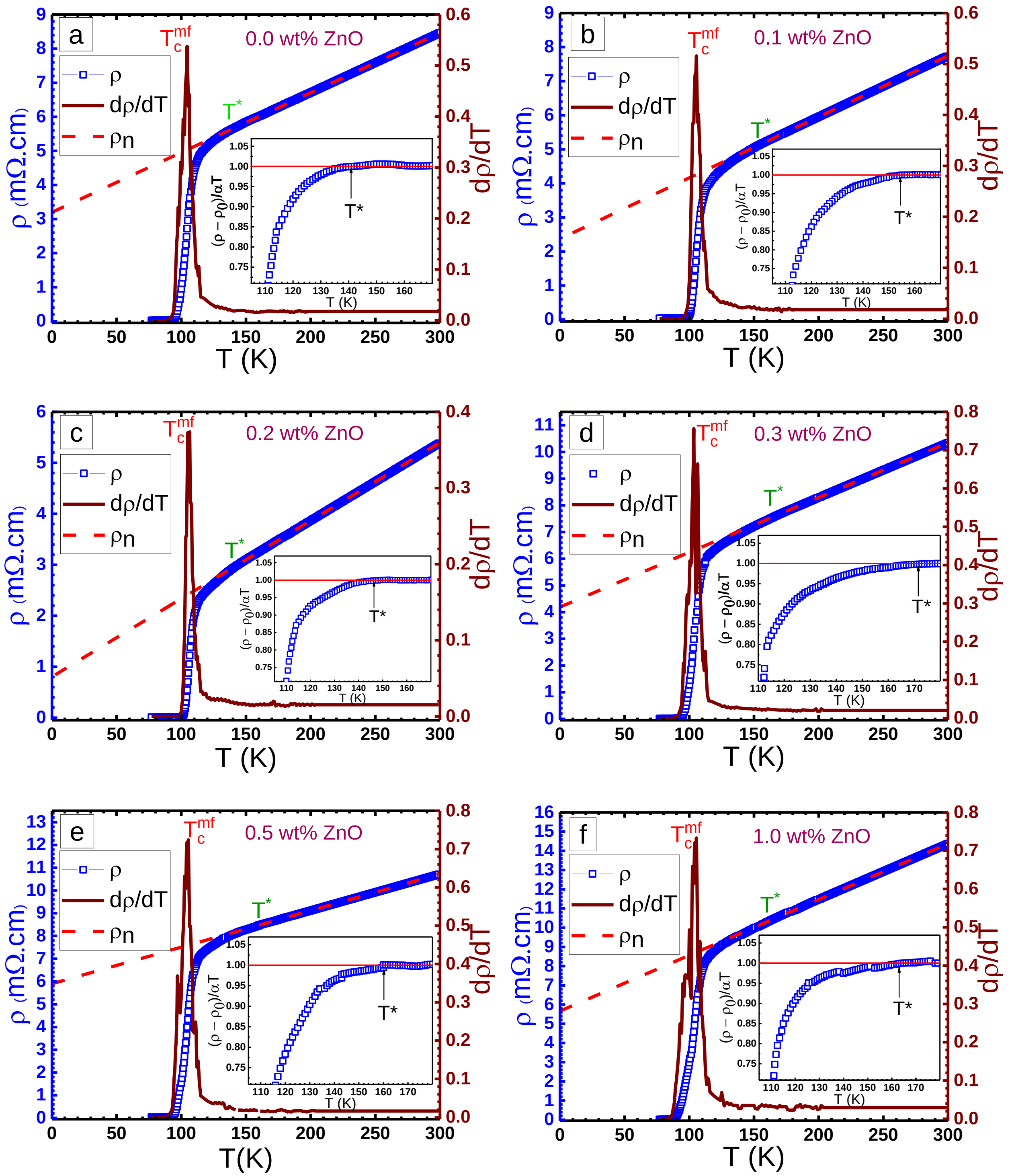

Fig. S1: Plots of the temperature dependence of electrical resistivity ρ(T), it's corresponding derivative dρ/dT, and extrapolated normal state resistivity $\rho_n(T)$ to 0 K, for different (Bi, Pb)-2223/(ZnO NPs)x composites. The inset graph displays a more accurate determination of T* using the criterion $(\rho(T)-\rho_0)/\alpha T=1$.

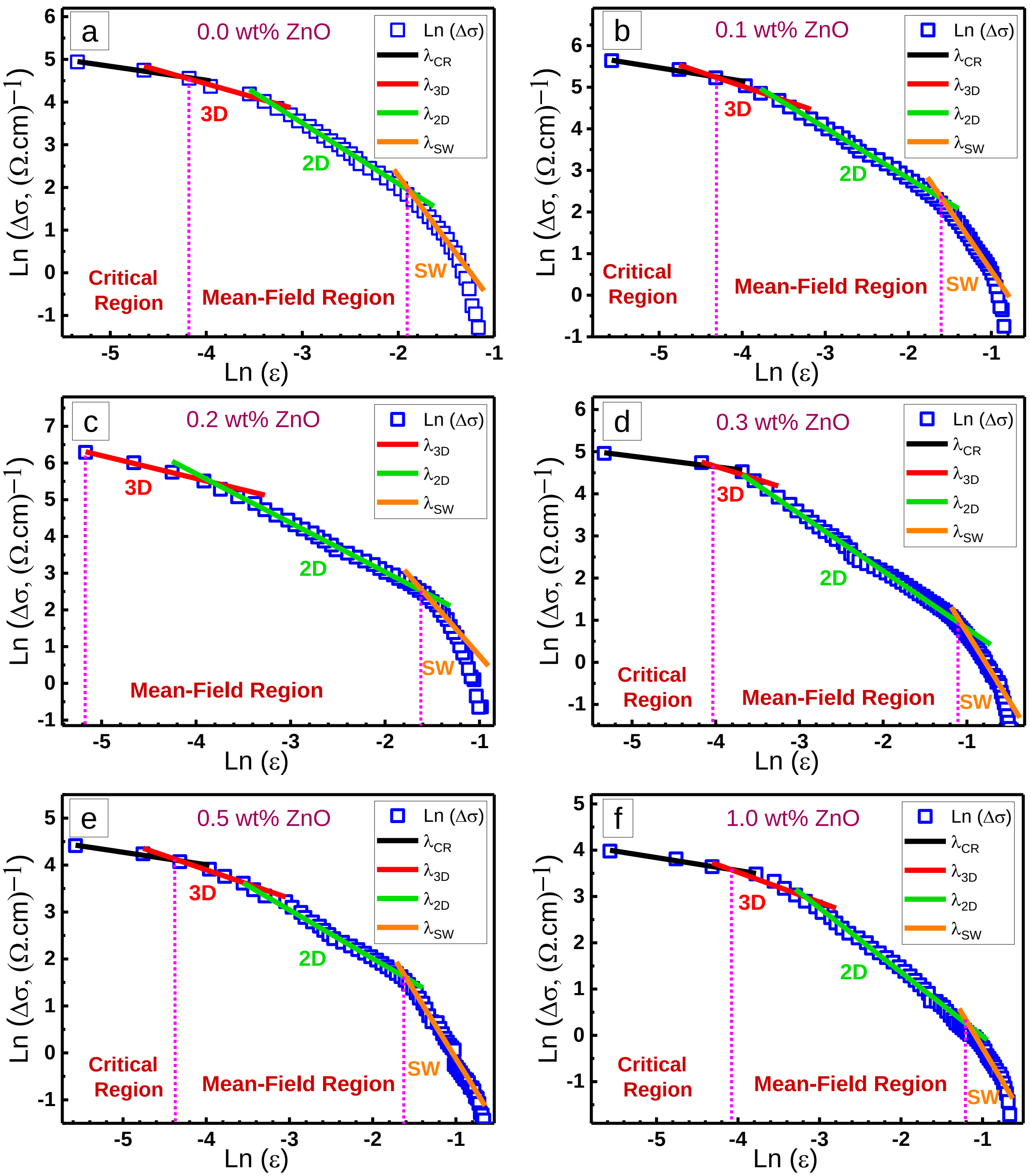

Fig. S2: The excess conductivity $\Delta\sigma$ as a function of reduced temperature ε in the Ln-Ln plot for different (Bi, Pb)-2223/(ZnO NPs)x composites. The solid lines show the fitting of the various fluctuation regions by the Aslamazov-Larkin theory.

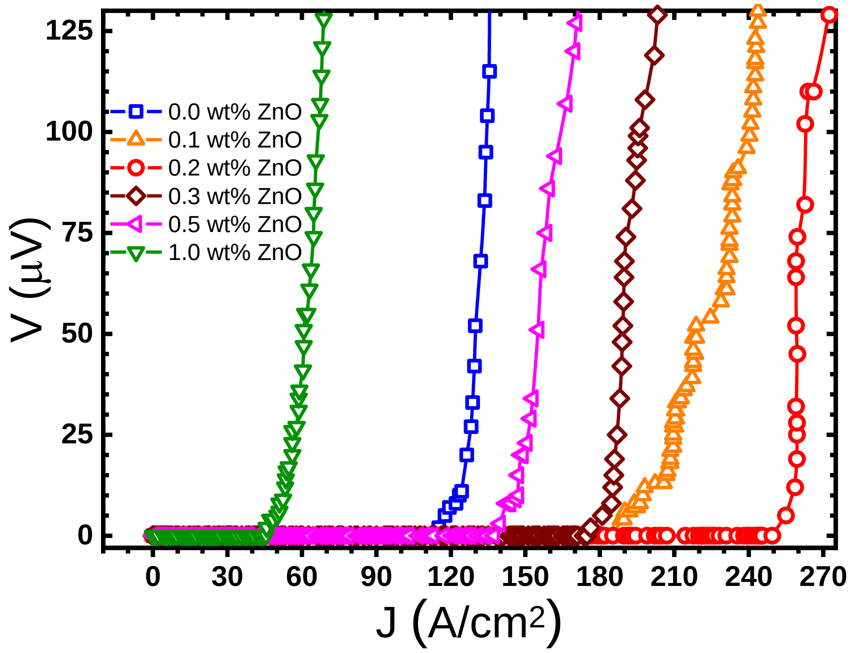

Fig. S3: V-J curves for different composites at 77 K.

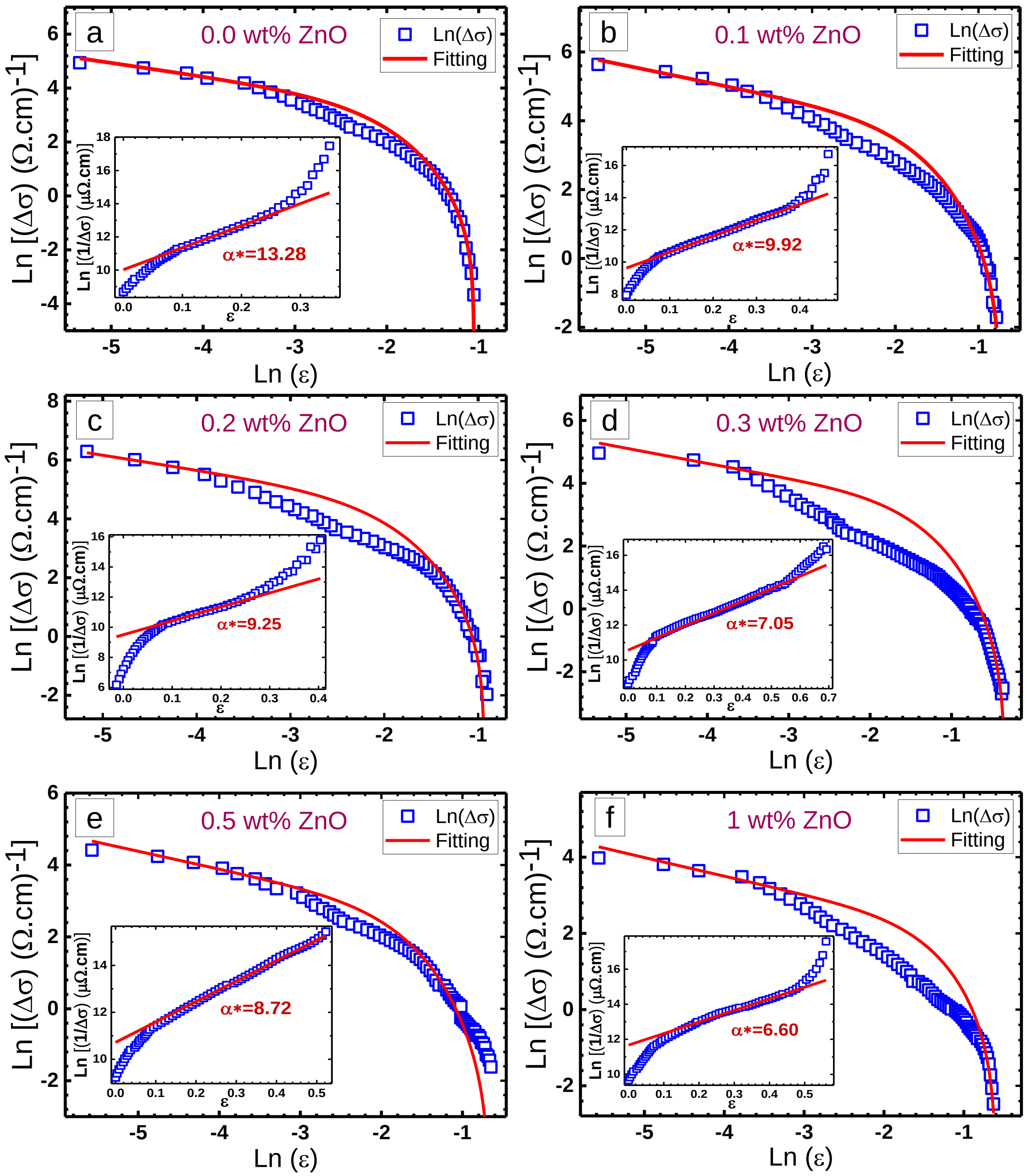

Fig. S4: Ln(Δσ) as a function of Ln(ε) (blue squares) for different (Bi, Pb)-2223/(ZnO NPs)x composites. The red solid curve is the fitting of the experimental data using the local pairs model (Eq. (14)). The inset graph shows Ln(1/Δσ) as a function of ε. The red solid line represents the fitting of the linear part of the curve above T_{2D-SW}. The inverse slope of which 1/α* determines the parameter ε_{co}^* ($\varepsilon_{co}^* = \frac{1}{\alpha^*}$).

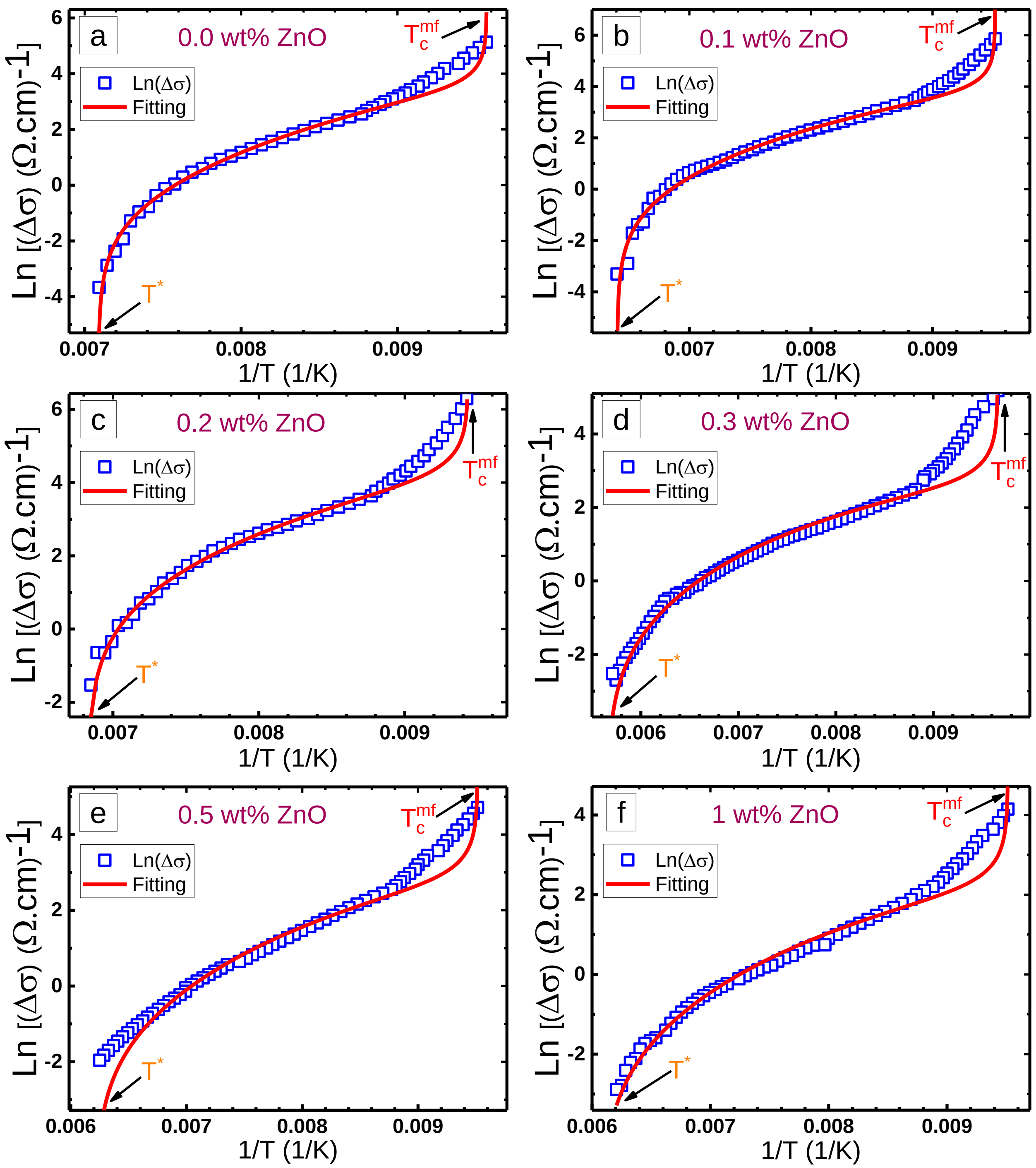

Fig. S5: Ln(Δσ) as a function of 1/T (blue squares) for different (Bi, Pb)-2223/(ZnO NPs)x composites. The red solid curve is the fitting of the experimental data using the local pairs model (Eq. (14)).
